# DNA damage protective effect of honey-sweetened cashew apple nectar in *Drosophila melanogaster*

**DOI:** 10.1590/1678-4685-GMB-2015-0129

**Published:** 2016-07-25

**Authors:** Robson Alves da Silva, Rafael Rodrigues Dihl, Lucas Pinheiro Dias, Maiane Papke Costa, Bianca Regina Ribas de Abreu, Kênya Silva Cunha, Mauricio Lehmann

**Affiliations:** 1 Laboratório de Toxicidade Genética (TOXIGEN), Programa de Pós-Graduação em Biologia Celular e Molecular Aplicada à Saúde (PPGBIOSAÚDE), Universidade Luterana do Brasil (ULBRA), Canoas, RS, Brazil; 2 Instituto Federal de Educação, Ciência e Tecnologia do Piauí (IFPI), Teresina, PI, Brazil; 3 Laboratório de Genética Toxicológica, Departamento de Bioquímica e Biologia Molecular, Instituto de Ciências Biológicas (ICB), Universidade Federal de Goiás (UFG), Goiânia, GO, Brazil

**Keywords:** antimutagenicity, cashew apple, Drosophila melanogaster, honey, somatic cells

## Abstract

Fruits and derivatives, such as juices, are complex mixtures of chemicals, some of which may have mutagenic and/or carcinogenic potential, while others may have antimutagenic and/or anticancer activities. The modulating effects of honey-sweetened cashew apple nectar (HSCAN), on somatic mutation and recombination induced by ethyl methanesulfonate (EMS) and mitomycin C (MMC) were evaluated with the wing spot test in *Drosophila melanogaster* using co- and post-treatment protocols. Additionally, the antimutagenic activity of two HSCAN components, cashew apple pulp and honey, in MMC-induced DNA damage was also investigated. HSCAN reduced the mutagenic activity of both EMS and MMC in the co-treatment protocol, but had a co-mutagenic effect when post-administered. Similar results were also observed with honey on MMC mutagenic activity. Cashew apple pulp was effective in exerting protective or enhancing effects on the MMC mutagenicity, depending on the administration protocol and concentration used. Overall, these results indicate that HSCAN, cashew apple and honey seem capable of modulating not only the events that precede the induced DNA damages, but also the *Drosophila* DNA repair processes involved in the correction of EMS and MMC-induced damages.

## Introduction

The ingestion of dietary components that decrease DNA damage accumulation may be an effective strategy for either the modulation or the inhibition of the carcinogenic process (Pingitore *et al.*, 2015). Hence, it is essential not only to assess safety and efficacy of candidate chemopreventive agents in preclinical models and in humans, but also to understand their mechanisms of action (Brown *et al.*, 2014; Liang and Kitts, 2014; Sloczynska *et al.*, 2014).

Fruits and their derivatives, such as juices, are complex mixtures of chemicals. Some of these substances may have mutagenic and/or carcinogenic potential, while others may diminish or abolish these effects (Bub *et al.*, 2003; Melo-Cavalcante *et al.*, 2003; Akeem *et al.*, 2011). Therefore, considerable attention has been given to the antimutagenic agents that occur naturally or are added to foods and beverages for human consumption (Ben Sghaier *et al.*, 2011). The formulation of mixed beverages or the addition of components to improve functional properties, such as cashew apple nectar sweetened with honey (Silva *et al.*, 2008; da Silva *et al.*, 2013), have been extensively studied (Akinwale, 2000; Jain and Khurdiya, 2004; Smith, 2006; de Sousa *et al.*, 2010).

A number of antimutagenic and anticancer substances that occur in nature, such as phenolic compounds, are found in cashew apple (Melo-Cavalcante *et al.*, 2003; Queiroz *et al.*, 2011; Bataglion *et al.*, 2015), honey (Gheldof *et al.*, 2002; Ferreira *et al.*, 2009; Baek *et al.*, 2015) and/or their derivatives. The beneficial effects of these compounds are partly attributed to their antioxidant activity.

In the present study, the *Drosophila* wing somatic mutation and recombination test (SMART), also known as the *Drosophila* wing-spot test, was used to evaluate the antimutagenic activity of honey-sweetened cashew apple nectar (HSCAN) on DNA damage induced by ethyl methanesulphonate (EMS) and mitomycin C (MMC), two known mutagenic compounds amply used as positive controls in antimutagenic studies (Santos *et al.*, 1999; Lehmann *et al.*, 2000; Sinigaglia *et al.*, 2004, 2006). Additionally, the protective activity of two components of HSCAN, cashew apple pulp and honey, was also evaluated. This bioassay allows the simultaneous detection and quantification of mitotic recombinations *versus* gene and chromosomal mutations (de Andrade *et al.*, 2004).

## Materials and Methods

### Chemicals

The mutagens ethyl methanesulphonate (EMS, CAS n° 62-50-0; Sigma-Aldrich, Saint Louis, MO, USA) and mitomycin C (MMC, CAS n° 50-07-5, Mitocin^®^, Bristol-Myers Squibb, Brazil) were dissolved in distilled water just before treatment. The mutagen doses used were established in previous pilot studies conducted in our laboratory to accurately evaluate antimutagenicity of HSCAN and other compounds. Drosophila Instant Medium Formula 4-24 was purchased from Carolina Biological Supply (Burlington, NC, USA) and powdered cellulose, from Merck (Darmstadt, Germany).

### Formulation of honey-sweetened cashew apple nectar (HSCAN)

The beverage HSCAN was prepared according to Silva *et al.* (2008), with 20% of cashew apple pulp, sweetened with honey to 11°Brix (measure of total soluble solids). It also contained sodium metabisulfite (40 ppm) and sodium benzoate (200 ppm) as stabilizers, (Vetec Química Fina Ltda., Duque de Caxias, RJ). This formulation was subjected to heat treatment at 90 °C for 1 min in an open stainless steel hot-bottling tank using a semi-automatic filler, and stored in 250 mL glass bottles sealed with plastic caps, which were subsequently cooled to 35 °C with tap water.

The cashew apple pulp frozen at -20 °C was obtained from a pulp mill located in the rural area of Teresina, Piauí, Brazil. The monofloral *Apis mellifera* honey (from floral cipó-uva; *Serjania sp*., Sapindaceae) was purchased at the local market in the city of Picos, Piauí, Brazil.

### Somatic Mutation and Recombination Test (SMART)

The standard (ST) cross flies were used for the wing spot test following the methods described in Graf *et al.* (1984): virgin females of the strain *flr* ^*3*^ */In(3LR)TM3, ri p* ^*p*^ *sep l(3)89Aa bx* ^*34e*^ *e Bd* ^*S*^ were crossed with *mwh/mwh* males. Information on the genetic markers is given by Lindsley and Zimm (1992).

Eggs were collected for 8 h in culture bottles containing a solid agar base (5% w/v agar agar in water) covered with a 5-mm layer of live baker's yeast supplemented with sucrose. After 3 days, the larvae were collected from these bottles with tap water strained through a fine-meshed stainless steel strainer and then used for the treatments.

All the experiments were carried out at 25 ± 1 °C and 60-70% relative humidity.

### Co-treatment

Three-day-old larvae were placed in equal batches into plastic vials containing 1.5 g of *Drosophila* Instant Medium and 5 mL of a test solution. EMS at 5 mM was tested alone or combined with HSCAN at 50 and 100%. MMC at 0.05 mM was tested alone or combined with HSCAN at 50 and 100%, cashew apple pulp at 20 and 100% and 73.0 mg/mL diluted honey solution. The genotoxins concentrations were based on previous studies (Abraham and Graf, 1996; Clements and Vogel, 1988; Santos *et al.*, 1999). For the negative controls, the larvae were transferred to a medium prepared with distilled water. Both control and treated larvae were allowed to feed on the medium for the rest of their development, which corresponds to approximately 48 h (Graf and van Schaik, 1992).

### Post-treatment

The 3-day-old larvae were initially distributed into plexiglass tubes, with bottom ends covered with fine nylon gauze. These tubes were then placed into 50-mL beakers containing 0.3 g of powdered cellulose added to 2 mL of distilled water or to a mutagen solution. The larvae fed through the gauze on water-cellulose solution, on 46 mM EMS-cellulose solution for 3 h, or on 0.12 mM MMC-cellulose solution for 6 h. The two groups submitted to acute feeding with water or genotoxins were then washed and transferred to several vials containing 1.5 g of *Drosophila* Instant Medium with either distilled water, or HSCAN (50 and 100%), cashew pulp (20 and 100%) and honey (73.0 mg/mL) solutions. The larvae were allowed to feed on the instant medium until pupation (± 48 h).

### Scoring of wings

The adult flies were collected on days 10 to 12 after treatments and stored in 70% ethanol. The ST cross produced two types of progeny that were distinguished phenotypically based on the *Bd* ^*S*^ marker: trans-heterozygous flies (*mwh/flr* ^3^) for the recessive wing-cell markers multiple wing hair (*mwh*) and flare (*flr* ^3^), and heterozygous flies (*mwh/TM3*) for a balancer chromosome with large inversions on chromosome 3 (*TM3*). Wings of females and males of the two genotypes were mounted on slides and scored under 400 magnification for the occurrence of spots. Induced loss of heterozygosity in the *mwh/flr* ^*3*^ genotype leads to two types of mutant clones: single spots, either *mwh* or *flr* ^3^, which result from point mutation, chromosome aberration, and/or mitotic recombination, and twin spots, consisting of both *mwh* and *flr* ^3^ subclones, which originate exclusively from mitotic recombination. In the *mwh/TM3* genotype, *mwh* spots reflect predominantly somatic point mutations and chromosome aberrations, since mitotic recombination involving the balancer chromosome and its structurally normal homologue is a lethal event (Vogel *et al.*, 1999). By comparing these two genotypes, it was possible to quantify the recombinagenic and mutagenic activities of each treatment (de Andrade *et al.*, 2004).

### Statistical analysis

Statistical assessment was done with the multiple-decision procedure of Frei and Würgler (1988), which allows four different results: positive, weakly positive, negative or inconclusive. The frequencies of each type of mutant clones per fly of a treated series were compared pair-wise (i.e., control *vs* genotoxin alone; genotoxin alone *vs* genotoxin plus HSCAN, cashew apple pulp and honey) using the conditional binomial test according to Kastembaum and Bowman (1970); *P <* 0.05 was considered significant. Because of the weak expression of the *flr* ^3^ marker in small clones and its lethality in large clones of mutant cells (Graf, 1995), only the *mwh* clones (*mwh* single and *mwh* twin spots) were used to calculate the clone formation frequencies per 10^5^ cells. These values were then employed to estimate the contribution of recombination and mutation to the incidence of total mutant spots per fly in trans-heterozygous flies (de Andrade *et al.*, 2004).

## Results

The analysis of both *mwh/flr* ^3^ and *mwh/TM3* genotypes allowed to evaluate the effect of co- and post-treatment with HSCAN on the genotoxicity of EMS (Table 1), and the effect of the treatment with HSCAN, cashew apple pulp and honey on the genotoxicity of MMC (Table 2). The data obtained in two individual experiments with each genotoxin (EMS and MMC) were pooled, since no statistical differences were found.

**Table 1:** Summary of results obtained in the wing spot test of *D. melanogaster* with co- and post-treatment series of EMS in combination with HSCAN.

Genotypes and treatments	No. of flies (*N*)	Spots per fly (no. of spots) statistical diagnosis^a^				Spots with *mwh* clone^c^(*n*)	Clone induction frequencies (per 10^5^ cells per cell division)^d^ (*n/NC*)^e^	
		Small single spots^b^	Large single spots^b^	Twin spots	Total spots^b^		Observed	Control corrected^f^
		(1-2 cells)	(> 2 cells)	*m = 5*	*m = 2*			
		*m = 2*	*m = 5*					
**Co-treatment**								
*mwh/flr* ^3^								
Negative control	50	0.58 (29)	0.12 (06)	0.08 (04)	0.78 (39)	39	1.60	
EMS 5 mM	50	28.58 (1429) *	12.36 (618) *	5.48 (274) *	46.42 (2321) *	2229	91.35	89.75
EMS + HSCAN 50%	50	28.06 (1403) -	11.74 (587) -	6.82 (341) w+	46 62 (2331) -	2217	90.86	89.26
EMS + HSCAN 100%	50	23.50 (1175) w+	8.96 (448) w+	5.64 (282) -	38.10 (1905) w+	1840	75.41	73.81
*mwh/TM3*								
Negative control	50	0.24 (12)	0.04 (02)	na	0.28 (14)	14	0.57	
EMS 5 mM	50	6.18 (309) *	2.34 (117) *	na	8.52 (426) *	426	17.46	16.89
EMS + HSCAN 50%	50	5.06 (253) w+	2.56 (128) -	na	7.62 (381) -	381	15.61	15.04
EMS + HSCAN 100%	50	4.20 (210) w+	1.36 (68) +	na	5.56 (278) w+	278	11.39	10.82
**Post-treatment**								
*mwh/flr* ^3^								
Negative control	40	0.55 (22)	0.25 (10)	0.10 (04)	0.90 (36)	36	1.84	
EMS 46 mM	40	3.40 (136) *	3.40 (136) *	1.40 (56) *	8.20 (328) *	304	15.57	13.73
EMS / HSCAN 50%	40	2.55 (102) w+	4.05 (162) -	2.25 (90) +	8.85 (354) -	336	17.21	15.37
EMS / HSCAN 100%	40	2.85 (114) -	5.60 (224) w+	2.35 (94) +	10.80 (432) w+	402	20.59	18.75
*mwh/TM3*								
Negative control	40	0.35 (14)	0.00 (00)	na	0.35 (14)	14	0.72	
EMS 46 mM	40	0.95 (38) *	0.55 (22) *	na	1.50 (60) *	60	3.07	2.36
EMS / HSCAN 50%	40	1.00 (40) -	0.65 (26) -	na	1.65 (66) -	66	3.38	2.66
EMS / HSCAN 100%	40	0.70 (28) i	0.90 (36) +	na	1.60 (64) -	64	3.28	2.56

Marker-trans-heterozygous flies (*mwh/flr* ^3^) and balancer-heterozygous flies (*mwh/TM3*) were evaluated.

a Statistical diagnoses according to Frei and Würgler (1988): *, positive; *P* ≤ 0.05 vs. untreated control. +, positive; w+, weakly positive; i, inconclusive; -, negative. *P* ≤ 0.05 vs. EMS alone.

b Including rare *flr* ^3^ single spots.

c Considering *mwh* clones from *mwh* single and twin spots.

d Calculated according to Frei *et al.* (1992).

e *C* = 48,800 (approximate number of cells examined per fly).

f Induction frequencies corrected for spontaneous incidence estimated from the negative controls. na: not applicable since only *mwh* single spots can be observed in *mwh/TM3* heterozygotes as the balancer chromosome *TM3* does not carry the *flr* ^3^ mutation.

**Table 2:** Summary of results obtained in the wing spot test of *D. melanogaster* with co- and post-treatment series of MMC in combination with HSCAN, pulp and honey.

Genotypes and treatments	No. of flies (*N*)	Spots per fly (no. of spots) statistical diagnosis^a^				Spots with *mwh* clone^c^(*n*)	Clone induction frequencies (per 10^5^ cells per cell division)^d^ (*n/NC*)^e^	
		Small single spots^b^	Large single spots^b^	Twin spots	Total spots^b^		Observed	Control corrected^f^
		(1-2 cells)	(> 2 cells)	*m = 5*	*m = 2*			
		*m = 2*	*m = 5*					
**Co-treatment**								
*mwh/flr* ^3^								
Negative control	40	0.45 (18)	0.20 (08)	0.05 (02)	0.70 (28)	28	1.43	
MMC 0.05 mM	40	22.08 (883) *	29.85 (1194) *	11.35 (108) *	63.28 (2531) *	2418	123.87	122.44
MMC + HSCAN 50%	40	16.33 (653) w+	22.43 (897) w+	8.93 (357) w+	47 68 (1907) w+	1830	93 75	92.32
MMC + HSCAN 100%	40	14.70 (588) w+	17.85 (714) w+	6.65 (266) w+	39.20 (1568) w+	1505	77.10	75.67
MMC + Pulp 20%	40	15.28 (611) w+	18.80 (752) w+	6.83 (273) w+	40.90 (1636) w+	1583	81 10	79.66
MMC + Pulp 100%	40	1.10 (44) +	0.80 (32) +	0.20 (08) +	2.10 (84) +	84	4.30	2.87
MMC + Honey^h^	40	18.78 (751) w+	23.03 (921) w+	7.95 (318) w+	49.75 (1990) w+	1914	98.05	96.62
*mwh/TM3*								
Negative control	40	0.20 (08)	0.00 (00)	na	0.20 (08)	08	0.41	
MMC 0.05 mM	40	6.83 (273) *	4.45 (178) *	na	11.28 (451) *	451	23.10	22.69
MMC + HSCAN 50%	40	5.45 (218) w+	3.10 (124) w+	na	8.55 (342) w+	342	17,52	17.11
MMC + HSCAN 100%	40	8.00 (320) w+	2.30 (92) +	na	10.30 (412) -	412	21.11	20.70
MMC + Pulp 20%	40	4.85 (194) w+	2.80 (112) w+	na	7.65 (306) w+	306	15.68	15.27
MMC + Pulp 100%	40	0.20 (08) +	0.10 (04) +	na	0.30 (12) +	12	0.61	0.20
MMC + Honey^h^	40	3.55 (142) +	2.40 (96) +	na	5.95 (238) +	238	12.19	11.78
**Post-treatment**								
*mwh/flr* ^3^								
Negative control	40	0.63 (25)	0.18 (07)	0.00 (00)	0.80 (32)	31	1.59	
MMC 0.12 mM	40	1.28 (51) *	7.50 (300) *	2.28 (91) *	11.05 (442) *	414	21.21	19.62
MMC / HSCAN 50%	40	0.83 (33) +	7.18 (287) -	2.70 (108) -	10.70 (428) -	406	20.80	19.21
MMC / HSCAN 100%	40	1.48 (59) -	7.68 (307) -	2.80 (112) -	11.95 (478) -	459	23.51	21.93
MMC / Pulp 20%	40	0.78 (31) +	5.18 (207) w+	2.10 (84) -	8.05 (322) w+	310	15.88	14.29
MMC /Pulp 100%	40	2.23 (89) +	7.58 (303) -	2.95 (118) w+	12.75 (510) w+	482	24.69	23.10
MMC / Honey^h^	40	2.45 (98) +	7.20 (288) -	4.50 (180)	14.15 (566) w+	548	28.07	26.49
*mwh/TM3*								
Negative control	40	0.40 (16)	0.05 (02)	na	0.45 (18)	18	0.92	
MMC 0.12 mM	40	0.50 (20) i	0.25 (10) *	na	0.75 (30) i	30	1.54	0.61
MMC / HSCAN 50%	40	0.15 (06) +	0.85 (34) +	na	1.00 (40) i	40	2.05	1.13
MMC / HSCAN 100%	40	1.00 (40) +	1.05 (42) +	na	2.05 (82) +	82	4.20	3.28
MMC / Pulp 20%	40	0.05 (02) +	0.60 (24) +	na	0.65 (26) -	26	1.33	0.41
MMC /Pulp 100%	40	0.30 (12) i	0.75 (30) +	na	1.05 (42) i	42	2.15	1.23
MMC / Honey^h^	40	0.50 (20) i	0.90 (36) +	na	1.40 (56) +	56	2.87	1.95

Marker-trans-heterozygous flies (*mwh/flr* ^3^) and balancer-heterozygous flies (*mwh/TM3*) were evaluated.

a Statistical diagnoses according to Frei and Würgler (1988): i, inconclusive; *, positive; *P* ≤ 0.05 vs. untreated control. +, positive; w+, weakly positive; -, negative; i, inconclusive. *P* ≤ 0.05 vs. EMS or MMC alone.

b Including rare *flr* ^3^ single spots.

c Considering *mwh* clones from *mwh* single and twin spots.

d Calculated according to Frei *et al.* (1992).

e *C* = 48,800 (approximate number of cells examined per fly).

f Induction frequencies corrected for spontaneous incidence estimated from the negative controls. na: not applicable since only *mwh* single spots can be observed in *mwh/TM3* heterozygotes as the balancer chromosome *TM3* does not carry the *flr* ^3^ mutation.

h 73.0 mg/mL.

### Positive controls

Tables 1 and 2 demonstrate that EMS and MMC were genotoxic and produced significant increases in all categories of spot in both genotypes compared to their respective negative controls. However, spot frequency induced in *mwh/TM3* flies was lower than those obtained in *mwh/flr* ^*3*^ genotype. These results are consistent with previously reported studies, confirming mitotic recombination as the prevalent genotoxic event induced by these genotoxins, using the SMART assay (Table 3) (Santos *et al.*, 1999; Sinigaglia *et al.*, 2004).

**Table 3:** Percentage of modulatory activity (inhibition or enhancement) and quantitative evaluation of mutation and recombination frequency to clone induction frequencies per 10^5^ cells per cell division.

Compounds and concentrations (mM)	Control corrected clone induction frequencies^a^ (↓ inhibition or ↑ enhancement)^b^		Recombination^c^ (%)	Mutation^c^ (%)
	*mwh/flr* ^3^	*mwh/TM3*		
**EMS**				
*Co-treatment*				
EMS 5 mM	89.75	16.89	81.19	18.81
EMS + HSCAN 50%	89.26	15.04	83.15	16.85
EMS + HSCAN 100%	73.81 (↓17.76%)	10.82 (↓35 94%)	85.34	14.66
*Post-treatment*				
EMS 46 mM	13.73	2.36	82.84	17.16
EMS / HSCAN 50%	15.37	2.66	82.67	17.33
EMS / HSCAN 100%	18.75 (↑ 36 56%)	2.56	86.34	13.66
**MMC**				
*Co-treatment*				
MMC 0.05 mM	122.44	22.69	81.46	18.54
MMC + HSCAN 50%	92.32 (↓24.60%)	17.11 (↓24.59%)	81.47	18.53
MMC + HSCAN 100%	75.67 (↓38.20%)	20.70	72.65	27.35
MMC + Pulp 20%	79.66 (↓34.94%)	15.27 (↓32.70%)	80.84	19.16
MMC + Pulp 100%	2.87 (↓97.66)	0.20 (↓99.12%)	92.86	7.14
MMC + Honey	96.62 (↓21.09%)	11.78 (↓ 48.08%)	87.80	12.20
*Post-treatment*				
MMC 0.05 mM	19.62	0.61	96.87	3.13
MMC / HSCAN 50%	19.21	1.13	94.13	5.87
MMC / HSCAN 100%	21.93	3.28 (↑437.70%)	85.05	14.95
MMC / Pulp 20%	14.29 (↓27.17%)	0.41	97.13	2.87
MMC / Pulp 100%	23.10 (↑17.74%)	1.23	94.68	5.32
MMC / Honey	26.49 (↑35.02%)	1.95 (↑219.67%)	92.65	7.35

a Calculated according to Frei *et al.* (1992).

e *C* = 48,800 (approximate number of cells examined per fly). Induction frequencies corrected for spontaneous incidence estimated from the negative controls.

b Inhibition (↓) or enhancement (↑) when compared to genotoxin alone. Calculated according to Abraham (1994): (genotoxin alone - genotoxin plus HSCAN, pulp or honey/genotoxin alone) x 100.

c Percentage of recombination (R) and mutation (M) were calculated according to Frei and Würgler (1996): R = 1 - [(n/NC in *mwh/TM3* flies) / (n/NC in *mwh/flr* ^3^ flies)] *100; M = 100 - R.

### Modulatory activity on EMS genotoxicity

The results for total spots show that the highest concentration of HSCAN modulated the genotoxic effect of EMS on both genotypes in the co-treatment, but only on *mwh/flr* ^3^ flies in the post-treatment (Table 1). The frequency of mutant clone formation in EMS-treated *mwh/flr* ^3^ and *mwh/TM3* flies decreased by ~18% and ~36%, respectively, after treatment with 100% HSCAN (Table 3). In this treatment the frequency of small and large single spots were also significantly reduced in both genotypes. In the 50% HSCAN-EMS co-treatment, an increase in twin spot frequency in *mwh/flr* ^*3*^ flies and a reduction in the number of small single spots in *mwh/TM3* genotype were also observed, but no significant alterations occurred in the other spot categories (Table 1).

Different results were observed when HSCAN was administered after the EMS-induced DNA damage. The 100% HSCAN was able to increase the frequency of total spots, large single spots and twin spots in trans-heterozygous flies, while a weak reduction of small single spots and an increase in twin spots was observed with 50% HSCAN, in this genotype. In *mwh/TM3* flies, no differences were found in any spot categories after post-treatment, with the exception of 100% HSCAN, which increased the incidence of large single spots.

Table 3 also shows the proportion of mitotic recombinations and somatic mutations calculated based on the clone induction frequencies per 10^5^ cells per cell division obtained for the two genotypes. For all treatments, mitotic recombination was the most prevalent mechanism involved in mutant clone induction. Additionally, the DNA damage reduction observed in 100% HSCAN co-treatment was related to mutational events, while the increased damage observed in post-treatment was related to recombination events.

### Modulatory activity on MMC genotoxicity

The results of the modulatory activity of HSCAN components cashew apple pulp and honey showed that all co-treatments were able to reduce the frequency of all spot categories in both genotypes, with the exception of total spots in 100% HSCAN treatment in *mwh/TM3* flies (Table 2). Although a weakly positive result was observed for almost all treatments, 100% cashew apple pulp produced a significant protective effect in the combined chronic treatments with MMC in *mwh/flr* ^3^ and *mwh/TM3* flies.

The decrease in clone induction frequency varied between 21.09% for honey and 97.66% for 100% cashew apple pulp in the *mwh/flr* ^3^ genotype, and from 24.59% for 50% HSCAN, to 99.12% for 100% cashew apple pulp in *mwh/TM3* flies (Table 3). With the exception of 100% HSCAN co-treatment, the other treatments reduced only DNA damage not related to recombinational events.

The response observed in the post-treatment protocols was quite different (Table 2). The frequency of total spots decreased only after 20% cashew apple pulp treatment, and HSCAN did not alter DNA damage incidence induced by MMC in the *mwh/flr* ^3^ genotype. On the contrary, a weak increase in total spot frequency was observed for 100% cashew apple pulp and honey post-treatments. In *mwh/TM3* flies, the incidence of total spots increased after treatments with 100% HSCAN and honey. No differences were observed in the other treatments, which presented inconclusive or negative results. Additionally, Table 2 shows that the incidence of small and large single spots, as well as of twin spots increased or diminished, depending on the treatment.

As shown in Table 3, although the 100% HSCAN post-treatment did not interfere with the incidence of total spots in *mwh/flr* ^3^ flies, it increased the incidence (437.70%) of DNA damage in the *mwh/TM3* genotype, which means that only DNA damage of mutational origin increased. The same effect was observed for the honey post-treatment, in which the increased spot frequency observed in the *mwh/TM3* genotype (219.67%) was much higher than that in the *mwh/flr* ^3^ genotype (35.02%). The opposite effect was observed for 20% and 100% cashew apple pulp post-treatments, i.e. reduction (27.17%) and increase (17.74%) in spot incidence, respectively, was seen only in trans-heterozygous flies, an outcome associated with recombinational events.

## Discussion

In the present study, the chemopreventive activity of HSCAN, cashew apple pulp and honey on DNA damage induced by EMS (an alkylating agent) and MMC (an alkylating and bifunctional DNA cross-linking agent) was investigated with the *Drosophila* wing SMART, using co- and post-treatment protocols. Marker-heterozygous (*mwh/flr* ^3^) and balancer-heterozygous (*mwh/TM3*) genotypes were analyzed, which allowed quantifying the contributions of recombination and mutation in the induced mutant spots. In a previous study by our research group, HSCAN and its constituents were assessed *in vitro*, to analyze their chemical properties and antioxidant potential, and *in vivo*, using the SMART to investigate their mutagenic activity. Chemical analysis showed that the pulp and HSCAN have high ascorbic acid concentrations; 277.09 and 67.22 mg/100 g, respectively. Additionally, 0.23 and 0.11 mg/100 g of carotenoids, 0.79 and 0.45 mg/100 g of anthocyanin and 61.10 and 10.90 mg/100 g of total phenolics were detected in the samples of pulp and HSCAN, respectively. In that study, DPPH and/or β-carotene/linoleate systems demonstrated a weak antioxidant capacity of honey, HSCAN and cashew apple pulp. The absence of mutagenic activity for HSCAN was observed using both standard and high-bioactivation cross of the SMART (da Silva *et al.*, 2013).

According to the present study, HSCAN, cashew apple pulp and honey modulate the mutagenic/recombinagenic activity of EMS and MMC, when administered in combination with or after the mutagens. HSCAN reduced the mutagenic activity of EMS and MMC in the co-treatment and increased the incidence of DNA damages induced by MMC and EMS in the post-treatment. The main components of HSCAN, cashew apple pulp and honey, were tested against the mutagenic activity of MMC only, and the results showed that cashew apple pulp displays a high antimutagenic profile when administered in the co-treatment protocol, at 20 and 100% concentrations. Moreover, it presented a co-recombinagenic activity at 100% concentration in post-treatment, and antirecombinagenic action, at 20% concentration. Similar results were found for honey co-treatment, which protected against MMC mutagenicity and, differently from cashew apple pulp, increased the frequency of mutations when post-administered.

The data herein suggest that HSCAN interferes with the preceding steps of DNA damage induced by EMS and MMC, acting as a desmutagenic compound. Moreover, it seems to interfere with the repair mechanisms involved in the MMC and EMS-induced DNA damage, increasing the incidence of mutations and recombinations, respectively. The same behavior was observed for honey and cashew apple pulp in relation to MMC.

These results are in line with literature, in which cashew apple juice was found to have mutagenic, radical-trapping, antimutagenic, and comutagenic activity (Melo-Cavalcante *et al.*, 2003, 2005, 2008, 2011; Spada *et al.*, 2008) using *in vitro* and *in vivo* bioassays. The Salmonella/microsome assay was employed to evaluate de antimutagenic activity of pre-, co- and post-treatments with cashew apple juice on mutations induced by aflatoxin B1(AFB1), methylmethanesulfonate (MMS), 4-nitroquinoline-N-oxide (4-NQO), benzo[a]pyrene (BaP) and hydrogen peroxide (Melo-Cavalcante *et al.*, 2003, 2005, 2008). AFB1-induced mutagenesis was suppressed in Salmonella strain TA102 when applied in co- and post-treatment, suggesting a modulatory activity on error-prone DNA repair and an interaction with S9 enzymes and metabolization to non-mutagenic compounds of AFB1 (Melo-Cavalcante *et al.*, 2005). In pre-treatment experiments with strains TA100 and TA102, fresh juice showed high antimutagenic activity against MMS but, conversely, co-treatment with fresh and processed juices enhanced MMS mutagenicity. In pre-, co-, and post-treatments with TA97a as test strain, antimutagenic effects were also observed against 4-NQO and BaP (Melo-Cavalcante *et al.*, 2008). A protective effect of cashew apple juice was likewise detected in the TA102 strain against mutation induced by hydrogen peroxide in co- and post-treatments. According to the authors, the antimutagenic effects during co-treatment could be associated with scavenging of free radicals and complex extracellular mutagenic compounds, while the protective effects observed in post-treatment may be due to stimulation of repair and/or reversion of DNA damage (Melo-Cavalcante *et al.*, 2003).

Mutagenic and antimutagenic evaluations with frozen cashew apple were also performed in eukaryotic cells of *Saccharomyces cerevisiae* yeast (Spada *et al.*, 2008). In that study, the frozen pulp showed mutagenic activity in three different concentrations (5, 10 and 15%) loci assayed in a dose-dependent manner, and did not display antimutagenic activity against hydrogen peroxide induced-mutations. Additionally, the antigenotoxic and anticlastogenic effects of cashew apple juice were assessed in the genotoxicity and mutagenicity induced by cyclophosphamide in male Swiss mice (Melo-Cavalcante *et al.*, 2011). The juice exerted antigenotoxic effects, decreasing the frequency of cyclophosphamide-induced micronucleus and DNA damage in peripheral blood of mice, and of chromosome aberrations in bone marrow.

Considering the high ascorbic acid concentrations found in cashew apple pulp and HSCAN, the observed antimutagenic and co-mutagenic effects might be a result of this vitamin's action. The antimutagenic activity of ascorbic acid was tested in the ST cross of the SMART in co-treatment experiments with potassium dichromate (K_2_Cr_2_O_7_), 4-nitroquinoline 1-oxide (4-NQO), and cobalt chloride (CoCl_2_) (Kaya *et al.*, 2002). Ascorbic acid was effective in reducing the genotoxicity of K_2_Cr_2_O_7_, but not of 4-NQO, at the same time that it caused significant increase in mutant clones when combined with CoCl_2_. In another investigation, the modulatory effect of ascorbic acid on the mutagenic activity of doxorubicin (DXR) was evaluated using the same assay, in both ST and high-bioactivation (HB) crosses (Fragiorge *et al.*, 2007). In that study, a protective effect was observed in the *mwh/flr* ^3^ genotype with the lowest concentration of ascorbic acid (50 mM), which was able to significantly decrease the frequency of spots induced by DXR (0.2 mM). On the contrary, an increased frequency of spots induced by DXR was observed with the highest concentration tested (100 mM). The authors suggested that ascorbic acid may interfere with DXR generated free radicals and with other possible reactive metabolites. This vitamin was also effective in reducing the incidence of micronucleus in human lymphocytes *in vitro* in the DXR co-treatment, but not in pre- and post-treatments (Amara-Mokrane *et al.*, 1996). Also on lymphocytes, cisplatin-induced clastogenesis was reduced by ascorbic acid in a co-treatment protocol (Lee, 2002). In another study with Drosophila, the X-chromosome linked recessive lethal mutation frequency induced by aflatoxin was reduced when this genotoxin was co-administered with ascorbic acid, and this mitigation could be based on the scavenging/trapping of DNA-reactive products by the antioxidant vitamin (Khan and Sinha, 2008). Additionally, ascorbic acid also reduced chromosome aberrations induced by rifampicin, an antituberculosis agent, when mice were co-treated with both compounds. Repeated doses of vitamin C reduced the percentage of rifampicin-induced DNA damage in a significant and dose-dependent manner (Aly and Donya, 2002). Bijur *et al.* (1997) suggest that the time at which vitamin C is administered in relation to oxidative stress exposure has an impact on its antimutagenic activity, which may explain the conflicting results concerning the effectiveness of ascorbic acid as a cancer chemopreventive agent.

Additionally, the other components found in HSCAN, as anthocyanins, carotenoids and phenolics, have also been characterized as antimutagenic compounds and could have influenced the observed results with HSCAN in the SMART assay. Mendoza-Díaz *et al.* (2012) observed that rich anthocyanins and carotenoids extracts from creole maize races were able to reduce the 2-aminoanthracene-induced mutagenicity on TA98 and TA100 strains of *Salmonella typhimurium*. Similarly, aqueous extracts of rose petals from different cultivars exhibited antimutagenicity against EMS-induced mutagenesis using the *Escherichia coli* RNA polymerase B forward mutation assay. The bioactive compound purified from the most potent rose cultivar was identified as an anthocyanin, which have potential health benefits (Kumar *et al.*, 2013). Among carotenoids, beta-carotene showed antigenotoxic properties in relation to radioactive iodine-131 and doxorubicin in Wistar rats using chromosomal aberration (Berti *et al.*, 2014) and micronucleus tests, respectively (Aissa *et al.*, 2012). The antimutagenic properties of different carotenoids from the green algae *Chlorococcum humicola* were observed in TA98, TA100 and TA102 strains of *S. typhimurium* with or without metabolic activation (Bhagavathy *et al.*, 2011). Monente *et al.* (2015) tested the main bioactive compounds in coffee extracts to determine their role in antimutagenic activity. The results indicated that 5-caffeoylquinic acid standard was highly effective in the inhibition of 4-nitro-o-phenylene-diamine (NPD) and 2-aminofluorene (2-AF) mutagens, mainly due to caffeic acid, which had similar antimutagenic activity.

Although honey has not been tested against EMS in the present study, it showed protective and co-mutagenic activity in co- and post-treatments, respectively, on MMC-induced DNA lesions. Previous chemical analysis of this honey sample by our research group, showed a total phenolic content of 31.91 mg of gallic acid equivalents (GAE) per 100 g, and a weak antioxidant capacity, when evaluated using the DPPH model system (da Silva *et al.*, 2013). Studies of the antimutagenic activity of honey are very scarce. However, different kinds of honey have been shown to protect DNA against physical and chemical mutagens, when evaluated using the *rpoB/*Rif^*R*^ test, the Yeast strain assay, the Salmonella/microsome test, and the chromosome aberration test in mice bone-marrow cells in co-treatment protocols, with different protective effects, depending on the geographical and floral sources (Wang *et al.*, 2002; Ezz El-Arab *et al.*, 2006; Guerrini *et al.*, 2009; Saxena *et al.*, 2012). The different effects of honey against induced mutagenesis could probably be due to differences in the samples' phenol and protein content (Saxena *et al.*, 2012). However, some authors found that sugars in honey contributed significantly to the antimutagenicity of this compound, and that monosaccharides were more potent antimutagens than disaccharides (Wang *et al.*, 2002).

Considering that the compounds tested in the present study are complex mixtures of bioactive components, the different antimutagenic responses found seem plausible. Moreover, these responses involve complex events, like the direct interaction with the genotoxin, antioxidative process, interactions with DNA repair mechanisms, and bioactivation/detoxification of metabolizing enzymes. In the co-treatment protocol, all the compounds were able to protect against the mutagenic activity of MMC, while HSCAN reduced also EMS mutagencity. Although MMC causes mainly DNA inter- and intrastrand cross-links and bulky 0^6^-guanine monoadducts, the protective effect against MMC may be associated with the antioxidant activity of compounds, as MMC is also able to generate free radicals (Turkez *et al.*, 2012). On the other hand, the preventive effect against EMS mutagenesis cannot be related to antioxidant activity, since EMS is an alkylating agent and a direct-acting mutagen. The protective action, therefore, is possibly mediated by the reaction of the tested compounds with reactive chemicals and trapping of the ethyl radical. The direct mispairing, caused by the addition of an EMS ethyl group to the O^6^ position of guanine and the O^4^ position of thymine in the DNA, could be scavenged by the reaction with some bioactive components of the honey, cashew apple pulp and HSCAN (Guerrini *et al.*, 2009).

Briefly, the data presented in this study show that HSCAN was able to reduce the mutagenic activity of EMS and MMC, when co-administered with these genotoxins, at the same time that it increased the frequency of mutagenic lesions when post-administered. The same behavior was observed for honey in relation to MMC genotoxicity. Cashew apple pulp presented different results only in the post-treatment protocol, in which protective and inducing activities were observed, depending on the applied concentration. In this sense, some studies have reported that bioactive components of fruits and vegetables can interfere with the expression of DNA repair enzymes (Ferguson *et al.*, 2015). Guarnieri *et al.* (2008) stated that lower levels of oxidized DNA following supplementation with phytochemicals suggest that the bioactive constituents may increase the activity of the DNA repair system in addition to a direct scavenging effect of reactive oxygen species.

Considering previously published data, a definitive conclusion about the protective effects of cashew apple pulp, honey and related beverages against chemical and physical DNA injuries cannot be drawn. Further studies with the *Drosophila* SMART assay and other bioassays should be performed to better understand the mechanisms and conditions underlying the chemopreventive and co-mutagenic activities of these compounds.
